# Magnetoelastic and Magnetoelectric Coupling in Two-Dimensional Nitride MXenes: A Density Functional Theory Study

**DOI:** 10.3390/nano13192644

**Published:** 2023-09-26

**Authors:** Sukhito Teh, Horng-Tay Jeng

**Affiliations:** 1 Department of Physics, National Tsing Hua University, Hsinchu 30013, Taiwan; 2 Institute of Physics, Academia Sinica, Taipei 11529, Taiwan; 3 Physics Division, National Center for Theoretical Sciences, Taipei 10617, Taiwan

**Keywords:** density functional theory, MXenes, multiferroic

## Abstract

Two-dimensional multiferroic (2D) materials have garnered significant attention due to their potential in high-density, low-power multistate storage and spintronics applications. MXenes, a class of 2D transition metal carbides and nitrides, were first discovered in 2011, and have become the focus of research in various disciplines. Our study, utilizing first-principles calculations, examines the lattice structures, and electronic and magnetic properties of nitride MXenes with intrinsic band gaps, including V_2_NF_2_, V_2_NO_2_, Cr_2_NF_2_, Mo_2_NO_2_, Mo_2_NF_2_, and Mn_2_NO_2_. These nitride MXenes exhibit orbital ordering, and in some cases the orbital ordering induces magnetoelastic coupling or magnetoelectric coupling. Most notably, Cr_2_NF_2_ is a ferroelastic material with a spiral magnetic ordered phase, and the spiral magnetization propagation vector is coupled with the direction of ferroelastic strain. The ferroelectric phase can exist as an excited state in V_2_NO_2_, Cr_2_NF_2_, and Mo_2_NF_2_, with their magnetic order being coupled with polar displacements through orbital ordering. Our results also suggest that similar magnetoelectric coupling effects persist in the Janus MXenes V_8_N_4_O_7_F, Cr_8_N_4_F_7_O, and Mo_8_N_4_F_7_O. Remarkably, different phases of Mo_8_N_4_F_7_O, characterized by orbital ordering rearrangements, can be switched by applying external strain or an external electric field. Overall, our theoretical findings suggest that nitride MXenes hold promise as 2D multiferroic materials.

## 1. Introduction

Intrinsic two-dimensional (2D) multiferroics have garnered significant attention in the research community due to their potential for downsizing and integration in various applications, allowing for the manipulation of ferroic orders through magnetoelectric, piezoelectric, or magnetoelastic interactions [1]. The experimental discovery of ferromagnetism in 2D CrI_3_ has spurred a rapid growth in fabricating 2D magnetic materials, including ferromagnets such as VI_3_ [2], CrBr_3_ [3], VSe_2_ [4], VTe_2_ [5], NbTe_2_ [6], MnSe_2_ [7], and Cr_2_Ge_2_Te_6_ [8,9]; and antiferromagnets such as CrCl_3_ [10], FePS_3_ [11], and MnPS_3_ [12], and exotic magnets such as α-RuCl_3_ [13], which exhibit Kitaev quantum spin liquid behavior. Additionally, numerous methods for controlling magnetization in 2D materials have been demonstrated, including electrostatic gate control of spin waves [14] and magnetic order [15,16] in bilayer CrI_3_, electrostatic gate control of the easy-magnetic axis in multilayer Cr_2_Ge_2_Te_6_ [17], and spin-current control of the magnetic order in CrSBr [18].

Considering their rich diversity in structure and composition, MXenes are promising 2D materials to realize multiferroicity. Tahir et al. [19] reported the first observation of ferroelectric and multiferroic properties in a delaminated Ti_3_C_2_T_x_ MXene film. Moreover, several theoretical studies have predicted intrinsic ferroelectricity or magnetism in some MXenes, such as i-MXene (Ta_2/3_Fe_1/3_)_2_CO_2_ as a type-I multiferroic material [20], Hf_2_VC_2_F_2_ as a type-II multiferroic material [21], and magnetic Janus MXenes, that can control their magnetic order via an out-of-plane electric field [22]. The recent advances in the synthesis of mono- or few-layer MXenes such as molten salt etching methods [23], halogen-based etching methods [24], the intercalation–alloying–expansion–microexplosion mechanism [25], and delamination based on the water freezing and thawing (FAT) method [26] have promised the production of large-lateral-size and high-quality MXenes that go beyond the well-studied Ti_3_C_2_T_x_. These new techniques give us the flexibility to modulate the composition of MXenes, including the control of functional group termination, and thus, examine the theoretical prediction of multiferroic properties in some MXenes. Nevertheless, there are only a limited number of reports concerning the fabrication of nitride MXenes, with the examples of V_2_NT_x_ [27], Mo_2_NT_x_ [27], Ti_2_NT_x_ [28], and Ti_4_N_3_T_x_ [29]. These reports primarily emphasize the fabrication processes of nitride MXenes and highlight their enhanced electrical conductivity compared to their carbide MXene counterparts. However, there is a notable absence of experimental studies regarding their magnetic properties and the possibility of multiferroicity within them.

Ab initio density functional theory (DFT) simulations can facilitate further exploration of 2D multiferroics for studying the electronic, magnetic, and mechanical properties of materials at a quantum mechanical level. Kumar et al. [30] carried out a systematic study on the magnetic properties of the nitride MXene M_2_NT_2_ and predicted that high-Curie-temperature ferromagnetism exists in multiple nitride MXenes, where M is an early transition metal (Ti, V, Cr, or Mn), N is Nitrogen, and T is a surface terminal group (O, OH, or F). Their results also hinted at the presence of orbital ordering in magnetic nitride MXenes, but did not fully elucidate its nature. Orbital ordering [31] is a common occurrence in oxides and plays important roles in many fascinating physical phenomena such as superconductivity [32,33,34,35], colossal magnetoresistance [36,37,38], and ferroelectric polarization [39]. Furthermore, it has been shown that electric fields can be used to control orbital ordering, Jahn–Tellar distortions [40], and the magnetic anisotropy of multiferroic PTO/LTO superlattices [41].

In this work, DFT simulations were used to investigate the structural, electronic, and magnetic properties of nitride MXenes M_2_NT_2_, with transition metals (M = V, Cr, Mn, or Mo), selected for their magnetic properties, and different surface terminations (T = O or F) chosen as functional groups to passivate the reactive transition metal surface. Our results show that the nitride MXenes V_2_NF_2_, V_2_NO_2_, Cr_2_NF_2_, Mo_2_NO_2_, Mo_2_NF_2_, and Mn_2_NO_2_ are semiconductors, with band gaps ranging from 0.20 eV to 2.1 eV, and exhibit intrinsic magnetism and orbital ordering. We found out that orbital ordering plays an important role in mediating magnetoelastic coupling in Cr_2_NF_2_ and magnetoelectric coupling in V_2_NO_2_ and the Janus MXene Mo_8_N_4_F_7_O.

## 2. Methods (Computational Approach)

### 2.1. Density Functional Theory

**Figure 1:**
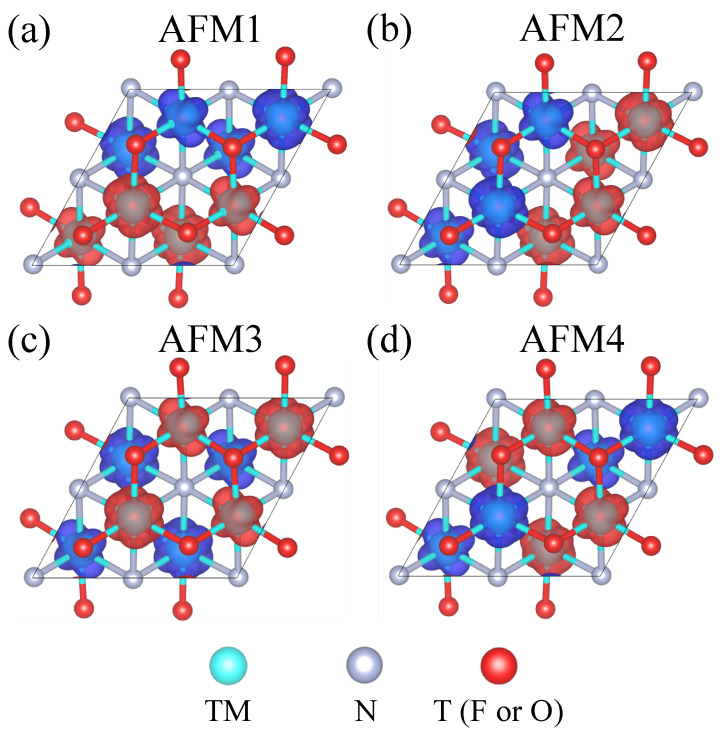
The spin densities of AFM patterns. Four AFM phases: (**a**) AFM1, (**b**) AFM2, (**c**) AFM3, and (**d**) AFM4 are considered in our calculations. Red and blue colors indicate up- and down-spin densities; the cyan, gray, and red spheres represent transition metals (TM), nitrogen, and terminal groups (oxygen or fluorine), respectively.

Density functional theory (DFT) calculations were performed using the Vienna ab initio simulation package (VASP) [42] with projector-augmented wave (PAW) pseudopotentials [43] and the Perdew–Burke–Ernzerhof (PBE) exchange-correlation functional [44]. Electron correlations in 3d and 4d electrons were treated using the rotational invariant on-site Coulomb parameters in Dudarev’s formulation [45]. Herein, the effective U values for V [46,47], Cr [46,48], Mn [46], and Mo [49,50,51] atoms are set to be 3 eV. A vacuum space of 20Å was used to prevent the interactions between the adjacent images along the vertical direction. Γ-centered 12×12×1 Monkhorst–Park k-point meshes were employed for structural optimizations and calculations of electronic properties, with the cutoff energy of the plane-wave basis set to 600 eV. To determine the ground state configuration, we performed structural optimization on the in-plane lattice constants and internal atomic coordinates of a 2 × 2 supercell with 5 different initial magnetic configurations, including four antiferromagnetic phases (AFM1, AFM2, AFM3, and AFM4 in Figure 1) and a ferromagnetic phase (FM), until the Hellman–Feynman force on each atom was less than $5\times 10^{-3}$ eV/Å. The nudged elastic band method [52] was used to determine the paths and energy barriers of ferroelectric switching, and ferroelectric polarization was calculated using the Berry phase method [53]. The ISOTROPY tool [54] was used to aid with the symmetry and space group analysis.

### 2.2. LKAG Formalism and Monte Carlo Simulations

To calculate the isotropic exchange parameters, $J_{ij}$, of two-dimensional nitride MXenes, we first derive the tight binding Hamiltonian based on the maximally localized Wannier function (MLWF) with the Wannier90 package [55,56], and then calculate the $J_{ij}$ by treating local spin rotation as a perturbation, using LKAG formalism [57] as implemented in the TB2J package [58]. These values of $J_{ij}$ were later used as the input parameters for the Monte Carlo (MC) simulations to verify the magnetic ground states and estimate the transition temperatures, using the Metropolis algorithm as implemented in the code SPIRIT [59]. The simulations adopted the Heisenberg spin model with a magnetic anisotropy term:

$$H=J_{ij}\sum_{ij}S_{i}\cdot S_{j}-A\sum_{i}{\left(S_{i}^{z}\right)}^{2}$$

with classical spin $S_{i,j}$ of length one at atomic sites *i*, *j*; single-ion magnetic anisotropy constant, *A*; component of the spin along the easy-magnetization axis, $S_{i}^{z}$. A 2D supercell of 50×100 (20,000 magnetic atoms) with a periodic boundary condition was adopted for the simulations, and for each temperature $2\times 10^{5}$ steps were taken for thermalization and another $2\times 10^{5}$ steps were performed for sampling.

## 3. Results and Discussion

### 3.1. Orbital Ordering, Electronic, and Magnetic Properties

**Figure 2:**
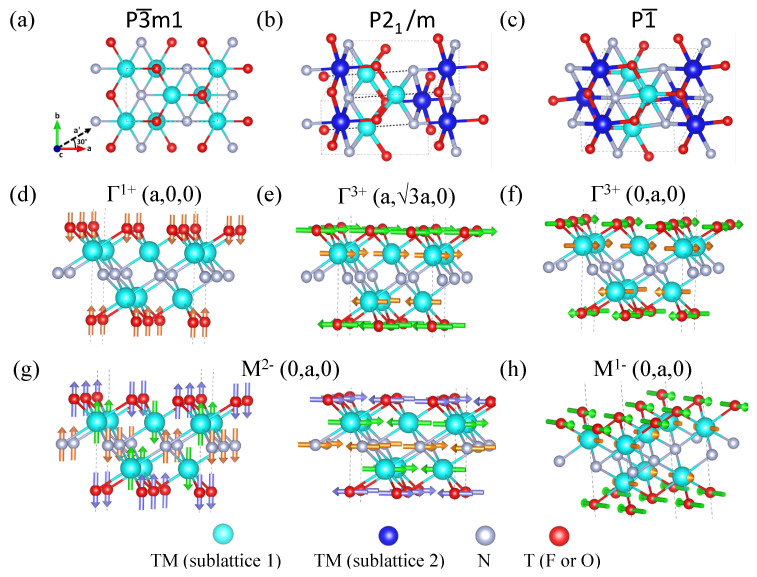
Lattice structure of space groups: (**a**) $P\bar{3}m1$, (**b**) $P2_{1}/m$, and (**c**) $P12_{1}/m1$. The cyan and dark blue spheres in (**b**,**c**) represent transition metals (TM) in sublattice 1 and sublattice 2, respectively. The distortion of the parent structure ($P\bar{3}m1$) can be decomposed into displacements with irreducible representations (**d**) $\Gamma_{1}^{+}\left(a,0,0\right)$, (**e**) $\Gamma_{3}^{+}\left(a,\sqrt{3}a,0\right)$, (**f**) $\Gamma_{3}^{+}\left(0,a,0\right)$, (**g**) $M_{2}^{-}\left(0,a,0\right)$, and (**h**) $M_{1}^{-}\left(0,a,0\right)$. The algorithm used to obtain these irreducible representations is summarized in Dorian and Harolds’ work [60].

**Figure 3:**
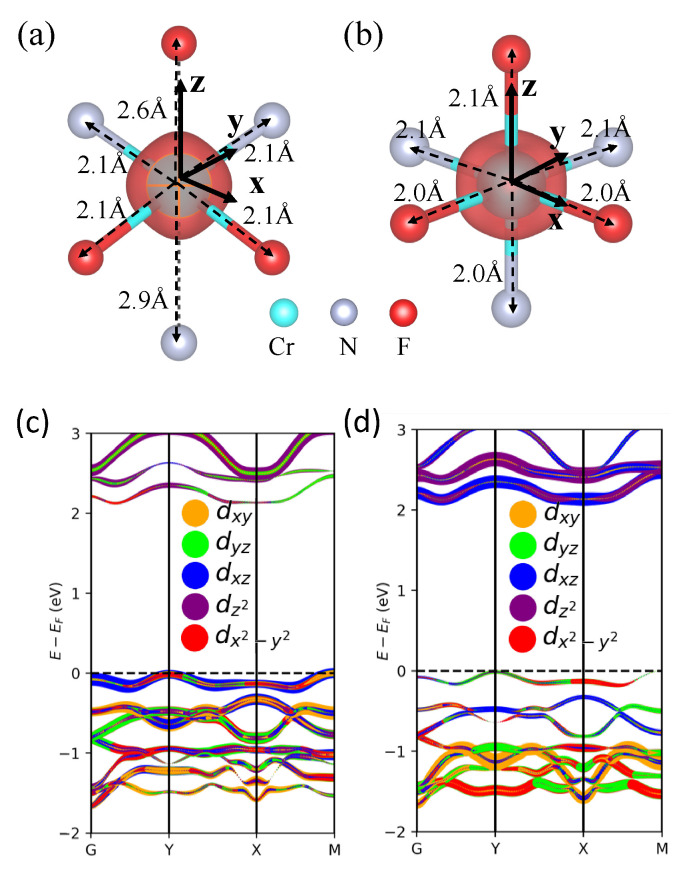
Contour plot of spin density in sublattices of Cr_2_NF_2_ with (**a**) lower ionization, sublattice 1, and (**b**) higher ionization, sublattice 2. The cyan, gray, and red spheres represent Cr, N, and F ions, respectively; the solid black arrows specify the axes of the local coordinate system for *d* orbitals. Projected band structure of Cr_2_NF_2_ on $d_{xy}$, $d_{yz}$, $d_{z^{2}}$, $d_{zx}$, and $d_{x^{2}-y^{2}}$ orbitals in (**c**) sublattice 1 and (**d**) sublattice 2.

The crystal symmetry analysis using ISOTROPY revealed that the space groups of the nitride MXenes V_2_NO_2_, CrNF_2_, MoNF_2_, and MnNO_2_ are monoclinic $P2_{1}/m$ (Figure 2b), while the space groups of V_2_NF_2_ and MoNO_2_ are triclinic $P\bar{1}$ (Figure 2c). Group theory analysis [60] using ISODISTORT shows that the distortion of the high-symmetry parent structure of space group $P\bar{3}m1$ (Figure 2a) into the $P2_{1}/m$ phase can be decomposed into three modes: the interplane antipolar displacements of T along the *c*-axis, with irreducible representation $\Gamma_{1}^{+}\left(a,0,0\right)$ (Figure 2d); the strain and interplane antipolar displacements of M and T along the *a*-axis, with irreducible representation $\Gamma_{3}^{+}\left(a,\sqrt{3}a,0\right)$ (Figure 2e); and the intraplane antipolar displacements of M, N, and T along either the *a*- or *c*-axis, with irreducible representation $M_{2}^{-}\left(0,a,0\right)$ (Figure 2g). In the $P\bar{3}m1$ parent phase, the M ions form an ideal octahedral complex with the X and T ions. The $\Gamma_{1}^{+}\left(a,0,0\right)$ displacements move the T ions closer to the M ions, thus shortening the M-T bonds; while $\Gamma_{3}^{+}\left(a,\sqrt{3}a,0\right)$ displacements move one T vertex closer to the central M ion and another two T vertices further from the central M ion, resulting in two longer M-T bonds and one shorter M-T bond in every octahedral complex. Finally, the $M_{2}^{-}\left(0,a,0\right)$ intraplane displacements shorten both M-X and M-T bonds in one metal complex while lengthening the M-X and M-T bonds in another, thus regrouping the M ions into two sublattices: sublattice 1 and sublattice 2 (Figure 2b). The M ions in sublattice 1 have longer average bond lengths and, thus, lower oxidation numbers, while sublattice 2 has shorter average bond lengths and higher oxidation numbers. Using Cr_2_NF_2_ as an example of structure $P2_{1}/m$, (Figure 3a,b) show that for both sublattice 1 and sublattice 2, the metal complexes have Jahn–Teller distortions, where the two axial bonds have different lengths when compared with the corresponding equatorial bonds.

The distortions of the $P\bar{1}$ structure can be decomposed into four modes: $\Gamma_{1}^{+}\left(a,0,0\right)$ mode (Figure 2d), $\Gamma_{3}^{+}\left(0,a,0\right)$ mode (Figure 2f), $M_{2}^{-}\left(0,a,0\right)$ mode (Figure 2g), and $M_{1}^{-}\left(0,a,0\right)$ mode (Figure 2h). The $\Gamma_{1}^{+}\left(a,0,0\right)$ mode and $M_{2}^{-}\left(0,a,0\right)$ mode are identical to their counterparts in the distortion of the $P\bar{1}$ structure, while the $\Gamma_{3}^{+}\left(0,a,0\right)$ mode’s antipolar displacements and strain are along the $a^{\prime }$-axis and this strain along the $a^{\prime }$-axis deforms the structure to a non-orthogonal cell. Other than that, there are additional intraplane antipolar displacements of M, N, and T along the *b*-axis with irreducible representation $M_{1}^{-}\left(0,a,0\right)$. The $M_{1}^{-}\left(0,a,0\right)$ intraplane displacements cause every metal–ligand bond in the octahedral complex to have different lengths.

**Figure 4:**
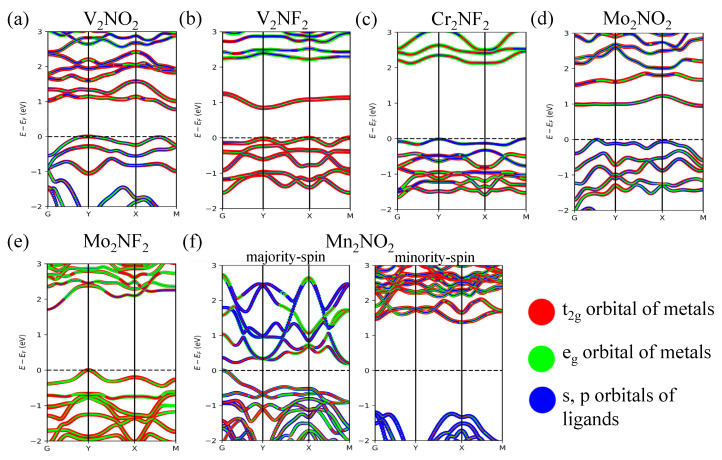
Projected band structures of (**a**) V_2_NO_2_, (**b**) V_2_NF_2_, (**c**) Cr_2_NF_2_, (**d**) Mo_2_NO_2_, (**e**) Mo_2_NF_2_, and (**f**) Mn_2_NO_2_ on the $t_{2g}$ and $e_{g}$ orbitals of metals M, and also on the *p* orbitals of the ligands (N, O, or F).

In order to better understand the electronic properties of the nitride MXenes, the ligand’s p-orbitals and the transition metal’s *d*-orbitals’ resolved band structures were plotted along high-symmetry paths in the Brillouin zone (Figure 4a–f). The results revealed that V_2_NF_2_, V_2_NO_2_, Cr_2_NF_2_, Mo_2_NO_2_, and Mo_2_NF_2_ exhibited the characteristics of a Mott transition, i.e., the valence band maximum (VBM) and conduction band minimum (CBM) were found to be primarily comprised of *d* electron bands. The band gap of these Mott insulating nitride MXenes is approximately 1 eV if the $t_{2g}$ orbitals are partially occupied, and approximately 2 eV for those with fully occupied $t_{2g}$ orbitals. In contrast, the band structure of Mn_2_NO_2_ shows a pd charge-transfer type of band gap, with the VBM being a mixture of $e_{g}$ orbitals from Mn and *p* orbitals from O, and the CBM dominated by $e_{g}$ and $t_{2g}$ orbitals.

Next, we analyze the nature of the orbital ordering by computing the magnetic properties. Table 1 summarizes the magnetic moments of metal ions (in nitride MXenes) at sublattice 1 and sublattice 2 sites, along with their ground state magnetic orders based on DFT calculations. The magnetic moments of the metal ions are calculated using Wannier functions based on the Wannier90 results of their respective magnetic ground states; as expected, the magnitudes of the magnetic moments of the metal ions are larger (smaller) at sublattice 1 (sublattice 2) as the number of occupied *d* orbitals is higher (lower). Since these transition metal complexes are in distorted octahedral or distorted tetrahedral symmetry, the *d* orbitals cannot be classified as pure $e_{g}$ or $t_{2g}$ orbitals, but rather a mixture of the two. For instance, the *d*-orbital occupancy of Cr^2+^ at the sublattice 1 of Cr_2_NF_2_ (Figure 3a) was found to be about three electrons per site, predominantly comprising a mixture of $d_{xy}$, $d_{yz}$, and $d_{zx}$ orbitals (Figure 3c). On the other hand, for Cr^3+^ at sublattice 2 (Figure 3b), the degeneracy of $e_{g}$ orbitals was lifted, with the $d_{z^{2}}$ orbital largely occupying the CBM; while the electronic states in the energy range of approximately 0.5 to 1 eV below the Fermi level were mainly occupied by $d_{xy}$, $d_{zx}$, and $d_{yz}$ orbitals (Figure 3d).

**Figure 5:**
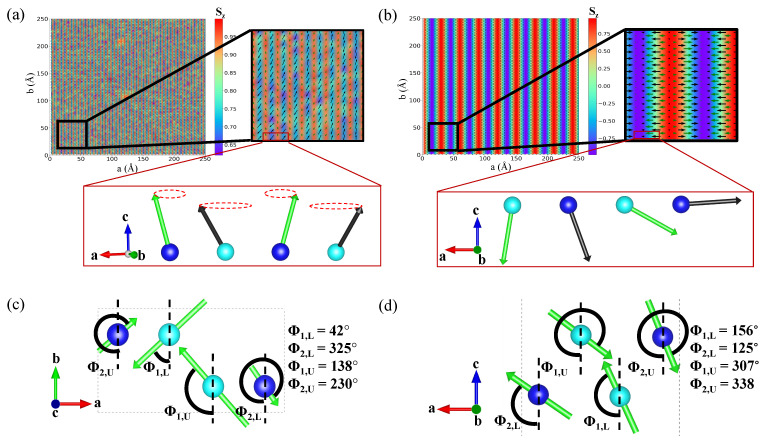
Spin textures of (**a**) Mn_2_NO_2_ at 0 K and (**b**) Cr_2_NF_2_ at 0 K generated using a Monte Carlo simulation in a 50 × 100 lattice with periodic boundaries. The color map is the magnitude of spin $S_{i}$ polarized along the out-of-plane axis; the green (black) arrows in sublattice 1 (2) represent the in-plane components of spin $S_{i}$; the cyan and blue spheres correspond to metal ions in sublattices 1 and 2, respectively. Spin components of (**c**) Mn_2_NO_2_ projected to a–b plane and (**d**) Cr_2_NF_2_ projected to a–c plane; $\phi_{i,j}$ represents the initial phase of the spin components in sublattice *i* in layer *j*.

Table 1 also summarizes the magnetic transition temperatures of the nitride MXenes as determined by Monte Carlo simulations. According to the Monte Carlo simulations, the magnetic ground states of V_2_NF_2_, V_2_NO_2_, Mo_2_NO_2_, and Mo_2_NF_2_ exhibit an AFM3 pattern, which agrees with the prediction of the DFT calculations. On the other hand, the Monte Carlo simulations predicted Cr_2_NF_2_ and Mn_2_NO_2_ to have non-collinear magnetic structures. The simulations revealed that Mn_2_NO_2_ displays a magnetic configuration similar to conical spin spiral (Figure 5a), with the magnetization at the atomic site *k* of sublattice *i* in layer *j*, with $R_{k(i,j)}$ approximated by $M_{k(i,j)}=M_{i}\left(\sin\left(\theta_{i}\right)\cos\left(q\cdot R_{k(i,j)}+\phi_{i,j}\right),\sin\left(\theta_{i}\right)\sin\left(q\cdot R_{k(i,j)}+\phi_{i,j}\right),\cos\left(\theta_{i}\right)\right)$, where $\theta_{1(2)}$≈ 43 (20)${}^{\circ }$ and $q\approx (\frac{\pi }{a},\frac{\pi }{b},0)$, with *a* and *b* representing the lengths of the lattice vector along the $\vec{a}$- and $\vec{b}$-axes, respectively. The initial phase of magnetization, $\phi_{i,j}$ is summarized in Figure 5c. It is worth noting that the long-range magnetic ordering in V_2_NF_2_, Mo_2_NO_2_, and Mo_2_NF_2_ is predicted to prevail above room temperature. Such room temperature magnetic behavior in nitride MXenes was also predicted by Kumar et al. [30], where Cr_2_NF_2_ and Mn_2_NT_2_ (T = F, O, and OH) are reported to show ferromagnetism, with Curie temperatures well above room temperature; pristine and La-doped Ti_3_C_2_ MXenes are reported to have ferromagnetism and antiferromagnetism coexisting at temperatures as high as 300 K [61]. Currently, most of the experimentally discovered 2D ferro-/antiferromagnets have their Curie/Néel temperatures limited below room temperature [2,3,5,6,7,8,9,10,11,12,13], with only VSe_2_ [4] and MnSe_x_ [7] showing strong ferromagnetism above 300 K. Our results and previous studies suggest that the MXenes are an important family of 2D materials to search for a new generation of room temperature ferro-/antiferromagnets.

**Figure 6:**
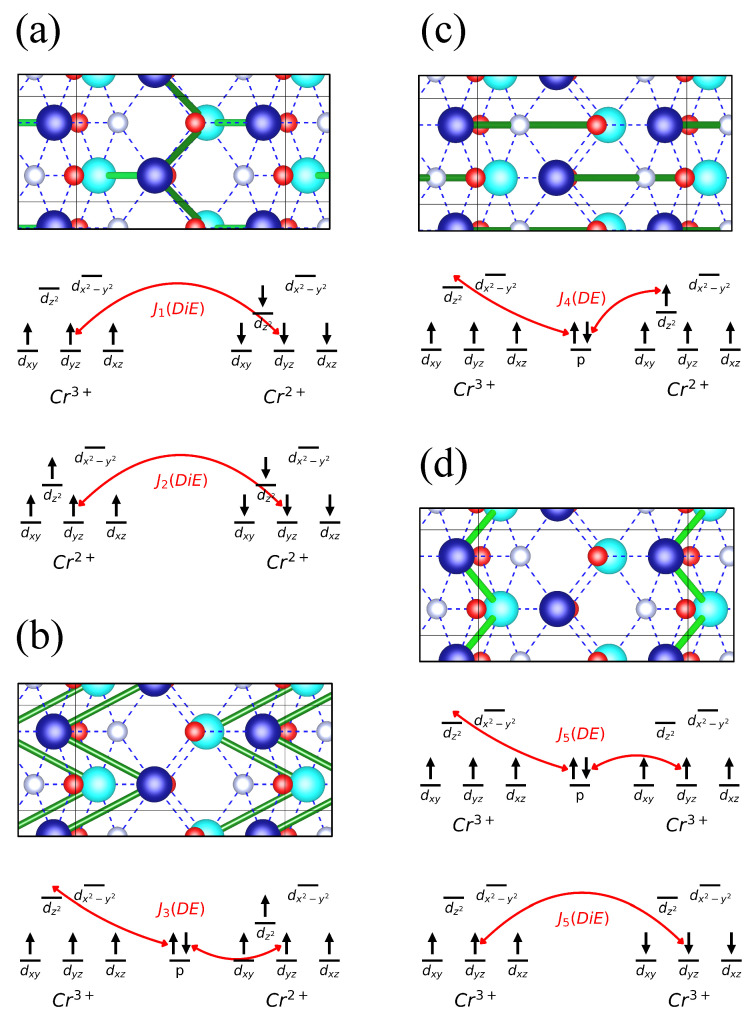
(**a**–**d**): Top view of Cr_2_NF_2_, where light blue, dark blue, gray, and red spheres correspond to chromium in the lower layer, chromium in the upper layer, nitrogen, and oxygen ions, respectively. The atomic bonds are represented in blue colored dashed lines, while green colored cylinders mark the Cr ion pairs with isotropic coupling: (**a**) J_1_ (dark green) and J_2_ (light green), (**b**) J_3_, (**c**) J_4_, and (**d**) J_5_.

For Cr_2_NF_2_, the Monte Carlo simulation at 0 K predicted a magnetic configuration (Figure 5b) that is similar to an out-of-plane Néel-type spin spiral, with the magnetization $M_{k(i,j)}=M_{i}\left(\cos\left(q\cdot R_{k(i,j)}+\phi_{i,j}\right),0,\sin\left(q\cdot R_{k(i,j)}+\phi_{i,j}\right)\right)$, where $q=(0.2\times \frac{2\pi }{a},0,0)$, where *a* represents the length of the lattice vector along the $\vec{a}$-axis. The formation of an intralayer spin spiral magnetization in Cr_2_NF_2_ is induced by frustrated isotropic couplings between Cr atoms, which can be described by a simplified model involving four values of the interatomic exchange coupling parameter J (Figure 6a–d). The strong antiferromagnetic couplings (J_1_ = −55 meV and J_2_ = −52 meV) between interlayer Cr atoms explain three key features of the Monte Carlo simulation results: (i) nearly antiparallel magnetization between Cr atoms from successive layers; (ii) nearly parallel magnetization between intralayer Cr atoms along the b-axis; and (iii) a small initial phase difference ($\Delta \phi \approx 30^{\circ }$) (Figure 5d) between intralayer Cr atoms within the same unit cell. The frustrated isotropic couplings are attributed to the competing interactions between intralayer ferromagnetic coupling J_3_ (18 meV) and interlayer ferromagnetic coupling J_4_ (28 meV), which favor ferromagnetic and antiferromagnetic couplings of intralayer Cr atoms along the a-axis and b-axis, respectively. Since magnetization of intralayer Cr atoms within the same unit cell are highly in phase, and the wavevector q is parallel to the a-axis, the value of q can be approximated using the classical J_N_-J_NN_ model for one-dimensional frustrated ferromagnets, i.e., q_a_ = $\left|J_{N}\right|$/(4J_NN_) [62]. Using J_NN_ = 2 × 18 meV and J_NN_ = 28 meV, we obtain q_a_ = 0.19, which is similar to the prediction of the Monte Carlo simulation (q_a_ = 0.20). Interestingly, although the interacting ion pairs of J_5_ are separated by comparable distances with similar atomic environments when compared with those of J_1_ and J_2_, J_5_ has a much weaker antiferromagnetic coupling (−4.4 meV) relative to J_1_ and J_2_. This unusual weak coupling is caused by orbital ordering in Cr_2_NF_2_, and is explained in detail in the next paragraph.

To understand the origin of antiferromagnetic and ferromagnetic interactions among Cr ions, we explain the magnetic interactions using two processes: the antiferromagnetic direct exchange process and the ferromagnetic double exchange process (Figure 6a–d). For J_1_ and J_2_, the interatomic distances between interacting ions are 2.772 Å and 3.149 Å, respectively, and the major contribution to the antiferromagnetic coupling originates from the direct exchange process between the Cr ions; the contribution from double exchange to J_1_ is negligible since the overlap between p-ligands and d-metals is lacking due to the non-occupancy of $d_{x^{2}-y^{2}}$ orbitals in metal ions; on the other hand, the distance between the connecting ligand and Cr ions in J_2_ coupling is too large, making double exchange processes unfavorable.

For the ferromagnetic J_3_ interaction, a double exchange process arises as the electron hops between the occupied $d_{yz}$ orbital in Cr^3+^ and unoccupied $d_{z^{2}}$ orbital in Cr^2+^, mediated by a non-magnetic N ion. While for the double exchange process in J_4_’s interaction, an electron from the occupied $d_{z^{2}}$ in Cr^2+^ couples with the unoccupied $d_{z^{2}}$ in Cr^3+^, through a non-magnetic N ion. Finally, a strong direct exchange process is expected in J_5_, as the separation between the interacting ions is only 2.946 Å. Nevertheless, a ferromagnetic double exchange process also arises from the electron coupling between the occupied $d_{yz}$ orbital in Cr^3+^ and unoccupied $d_{z^{2}}$ orbital in Cr^3+^ at the opposite site. The competing interaction between the double exchange process and direct exchange process results in the weak coupling strength of J_5_ (−4.4 meV).

### 3.2. Magnetoelastic Coupling in Cr_2_NF_2_

**Figure 7:**
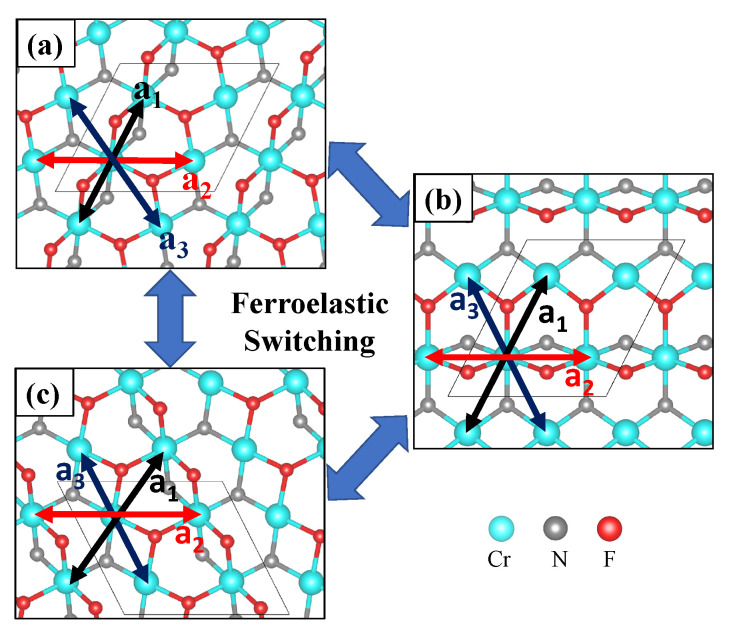
Ferroelastic transition of Cr_2_NF_2_. Top view of lower layer at (**a**) ground state with $a_{2}$ = $a_{3}$ > $a_{1}$, (**b**) ground state with $a_{1}$ = $a_{3}$ > $a_{2}$, and (**c**) ground state with $a_{1}$ = $a_{2}$ > $a_{3}$.

The material Cr_2_NF_2_ possesses a significant ferroelastic strain (10.9%), i.e., it has three equally stable orientation variants in the lattice structure that can be switched by applying an external stress. The ferroelastic strain is defined as $\left(\left(\frac{b}{a}-1\right)\times 100\%\right)$, with lattice parameters a=6.03 Å and b=6.689 Å, calculated along the three diagonal lines ($\vec{a_{1}},\vec{a_{2}}$, and $\vec{a_{3}}$) of a deformed hexagon (Figure 7a). The shorter lattice parameter, *a*, is laying along $\vec{a_{1}}$, and the propagation vector of the spin spiral magnetization is perpendicular with respect to $\vec{a_{1}}$, showing coupling between the ferroelastic strain and magnetic order.

In addition to the previously mentioned orientation state (Figure 7a), there are two equivalent orientation states that can be achieved in Cr_2_NF_2_, by switching the values of $a_{2}$ (Figure 7b) or $a_{3}$ (Figure 7c) to 6.03 Å. The transitions between the two orientation states are analyzed using the nudged elastic band (NEB) method and result in an activation energy barrier of 56 meV per atom. In comparison to other reported 2D ferroelastic materials, Cr_2_NF_2_ has a low ferroelastic strain relative to BP_5_ (41.4%) [63], borophane (42%) [64], and phosphorene (37.9%) [65], but is similar to AgF_2_ (13.5%) [66] and t-YN (14.4%) [67]. The activation energy barrier of Cr_2_NF_2_ is also moderate, comparable to AgF_2_ (51 meV) [66] and t-YN (33 meV) [67], but lower than that of BP_5_ (320 meV) [63], borophane (100 meV) [64], and phosphorene (200 meV) [65]. These results suggest that the moderate activation energy barrier of Cr_2_NF_2_ allows for experimental manipulation of its ferroelasticity while maintaining stability, which could facilitate the control of its spin spiral magnetization.

### 3.3. Orbital-Ordering-Induced Magnetoelectric Coupling

It is noteworthy that structural optimization has revealed the existence of stabilized ferroelectric (FE) phases in the materials V_2_NO_2_, Cr_2_NF_2_, and Mo_2_NF_2_. These FE phases are characterized by energies that are 31 meV, 70 meV, and 47 meV higher than the energies of their corresponding ground states, respectively.

**Figure 8:**
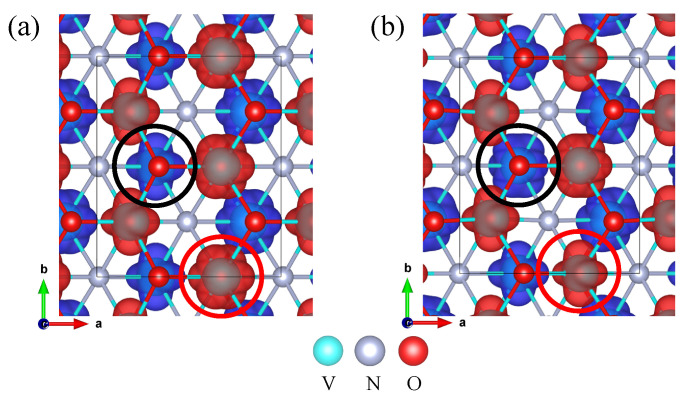
Contour plot of spin density of (**a**) antiferroelctric phase (ground state), (**b**) ferroelectric phase of V_2_NO_2_; where the yellow and blue colored isosurfaces represent spin-up and spin-down density, respectively. The V ion in the lower (upper) layer circled in black (red) is referred to as V_L_ (V_U_).

As an illustration, we use V_2_NO_2_ to highlight the role of orbital ordering in magnetoelectric coupling among the aforementioned MXenes. Figure 8a,b present contour plots of the spin density of V_2_NO_2_ in the antiferroelectric (AFE) phase (ground state) and FE phase (excited state), respectively. Both phases exhibit AFM3 orders. The transition from the AFE phase to the FE phase results in a significant increase in the average metal–ligand bond length of the V_L_ ion (circled in black in Figure 8a,b) in the lower layer and a decrease in the average metal–ligand bond length of the V_U_ ion (circled in red in Figure 8a,b) in the upper layer. This in turn leads to the transformation of V_L_ and V_U_ ions from V^4+^ and V^3+^ to V^3+^ and V^4+^, respectively. As a consequence, the distribution of V^4+^ and V^3+^ ions becomes asymmetrical, with the ratio of V^4+^ to V^3+^ being 1:3 and 3:1 in the upper and lower layers of V_2_NO_2_, respectively.

To further verify the stability of the AFM3 order of V_2_NO_2_, we compared the energies of the FE phase at different magnetic orders and performed Monte Carlo simulations. The results confirmed that the AFM3 order remains the most stable magnetic configuration. The energy required to switch from the AFE to the FE phase is estimated to be 51 meV per V atom using the nudged elastic band (NEB) method. The Berry phase method calculates the polarization along the a- and c-axes to be 6.9 µC/cm^2^ and 2.3 µC/cm^2^, respectively, expressed in bulk form by considering its thickness. Since the magnetic moment of V^3+^ (≈2 µ${}_{B}$) is larger than that of V^4+^ (≈1 µ${}_{B}$), the asymmetrical distribution of V^4+^ and V^3+^ ions in the FE phase results in a net average magnetic moment of 0.25 µ${}_{B}$ per V atom, demonstrating the coupling between the electrical and magnetic degrees of freedom.

Nevertheless, the excitation energy (32 meV) of the ferroelectric phase of V_2_NO_2_ is likely too high to be stabilized experimentally. One potential method to induce spontaneous ferroelectric polarization is to fabricate Janus MXenes, which are MXene structures with different compositions of the upper and lower functional groups. The absence of inversion symmetry in Janus MXenes leads to a large dipole moment. However, this type of ferroelectric polarization usually cannot be switched by an external electric field, which limits its application in nanoelectronic devices.

**Figure 9:**
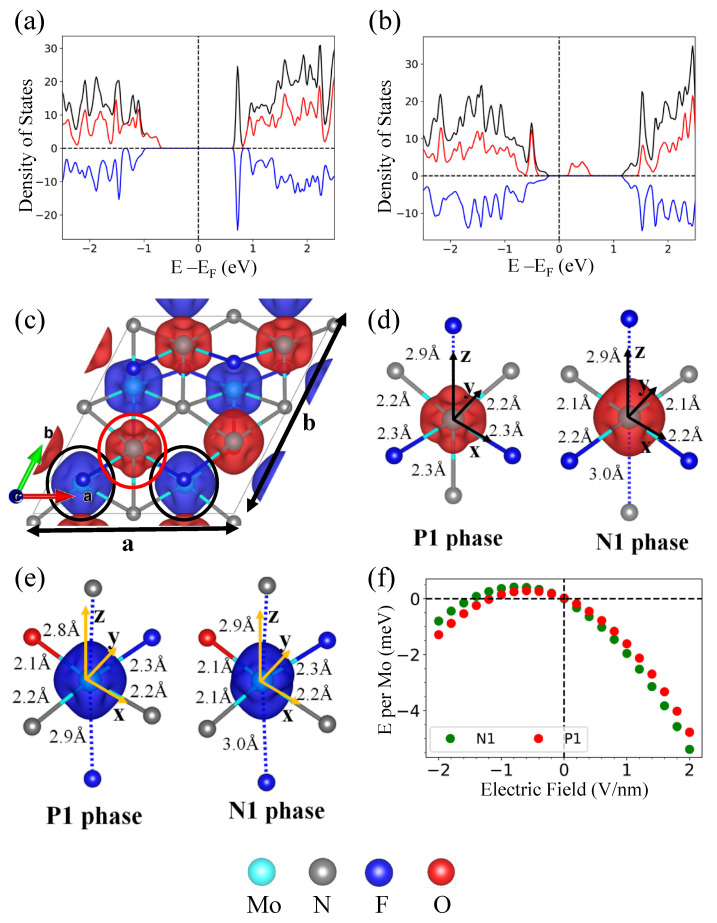
The density of states of Mo_8_N_4_F_7_O in (**a**) P1 phase and (**b**) N1 phase. (**c**) Contour plot of spin density of Mo_8_N_4_F_7_O in P1 phase with ferroelastic strain of 10.2%, where the blue and red colored isosurfaces represent spin-up and spin-down density, respectively. The Mo ion circled in black (red) is referred as Mo_L_ (Mo_U_), where (**d**,**e**) illustrates the change in orbital ordering of Mo_U_ and Mo_L_ during the transition from the P1 to the N1 phase, respectively. (**f**) Energy of Mo_8_N_4_F_7_O in the P1 and N1 phases as a function of applied electric field in the out-of-plane direction at the ferroelastic strain of 10.2%.

In this study, we demonstrate that ferroelectric polarization and magnetization in the Janus MXene Mo_8_N_4_F_7_O can be altered by an external electric field or external strain. We found out that the Janus MXenes V_8_N_4_O_7_F, Cr_8_N_4_F_7_O, and Mo_8_N_4_F_7_O have excited states characterized by orbital ordering rearrangements, and the excitation energies of these Janus MXenes (18.5 meV, 18.6 meV, and 6.6 meV, respectively) are much lower than those of their non-Janus counterparts, i.e., V_2_NO_2_, Cr_2_NF_2_, and Mo_2_NF_2_. Interesting results are found in the transition of Mo_8_N_4_F_7_O from the ground state phase (P1 phase) to the excited phase (N1 phase). Our results reveal that Mo_8_N_4_F_7_O is a bipolar magnetic semiconductor (BMS) in both the P1 and N1 phases. BMSs are characterized by a VBM and CBM that are fully spin polarized in opposite spin channels (Figure 9a,b), offering an ideal platform to manipulate the polarization of spin current using gate voltage [52,68,69]. In the P1 (N1) phase, the VBM is dominated by electronic contributions from lower (upper)-layer Mo ions, while the CBM is dominated by electronic contributions from upper (lower)-layer Mo ions, with a band gap of 1.4 (0.4) eV. The transition from the P1 to N1 phase also results in an increase in the ferroelastic strain, $(\frac{b}{a}-1)\times 100\%$ (Figure 9c), from 6.0% to 13.1%, with $a_{P1}=6.57$ Å and $b_{P1}=6.96$ Å, while $a_{N1}=6.38$ Å and $b_{N1}=7.22$ Å.

By linearly interpolating *a* and *b* between ($a_{P1}$,$b_{P1}$) and ($a_{N1}$,$b_{N1}$), we are able to further reduce the excitation energy until the N1 and P1 phases become nearly energetically degenerate (around a ferroelastic strain of 10.2%). To illustrate the orbital ordering rearrangements upon the transition, we show the (contour plot of) spin density of the P1 phase (Figure 9c) and highlight the changes upon transition (Figure 9d,e). In the P1 phase, the Mo atoms in the lower layer (75% F and 25% O as surface functional groups) have a higher average magnetic moment than the Mo atoms in the upper layer (with only F as a surface functional group), thus give a net magnetic moment of +0.25 µ${}_{B}$ per Mo. Upon transition to the N1 phase, the bond length between Mo_U_ (circled in red in Figure 9c) and the nearest N ion along the negative z-direction (of local coordinate system for d-orbitals) increases significantly, thereby increasing the occupancy of the $d_{z^{2}}$ orbital and magnetic moment at the Mo_U_ site; since the N ion now receives less electrons from the Mo_U_ site, Mo_L_ ions (circled in black in Figure 9c) donate additional electrons to the N ion, resulting in a slight decrease in occupancy of $d_{xy}$ orbitals and the magnetic moments at the Mo_L_ site. As a result, the transition from the P1 phase to N1 phase changes the average magnetic moment from +0.25 µ${}_{B}$ per Mo to −0.25 µ${}_{B}$ per Mo.

Because of the broken inversion symmetry in Mo_8_N_4_F_7_O and the difference in electronegativity of O and F, we expect an intrinsic out-of-plane electric field directed from the lower layer to the upper layer. This prediction is confirmed by our calculations of electronic energies under an applied electric field (Figure 9f): both P1 and N1 phases have their electronic energies decrease (increase), with the applied electric field directing upward (downward). However, the electronic energy of the N1 phase decreases more significantly with an upward applied electric field, and increases less significantly with a downward electric field, when compared with the energy of the P1 phase under the same conditions, implying that the overall strength of the intrinsic electric field is higher in the N1 phase relative to the P1 phase. From Figure 9c, we can see that in the P1 phase, the total ionic charge of Mo ions from the upper layer is higher than from those in the lower layer; such an asymmetrical distribution of low and high ionic charge on the Mo ions results in an intrinsic out-of-plane electric field that is opposite to the intrinsic electric field due to the asymmetrical surface functional groups, thus reducing the overall strength of the intrinsic electric field. On the other hand, the intrinsic electric field, due to both the asymmetrical distribution of low/high-ionic-charge Mo ions and of surface functional groups, is pointing upwards, thus enhancing the overall strength of the intrinsic electric field. The results show that at a ferroelastic strain of 10.2%, the energies of Mo_8_N_4_F_7_O in the P1 and N1 phases become degenerate, where switching of the phase can be achieved by application of an external electric field, making non-volatile electric control of spin polarization possible. It should be noted that the origin of magnetoelectric coupling in our study is due to orbital ordering, which is similar to the previously reported multiferroicity in LuFe_2_O_4_ [70]. In LuFe_2_O_4_, the non-centrosymmetric distribution of Fe^2+^ and Fe^3+^ would give rise to spontaneous ferroelectricity and ferrimagnetism. To quantify the coupling between the magnetic order, electronic polarization, ionic displacement, and lattice distortion, a detailed study using a Landau-like model [71,72] is required in the future.

## 4. Conclusions

In conclusion, using first-principles calculations, we have shown that multiple nitride MXenes (V_2_NO_2_, V_2_NF_2_, Cr_2_NF_2_, Mo_2_NO_2_, Mo_2_NF_2_, Mn_2_NO_2_) are magnetic semiconductors, with band gaps ranging from 0.20 eV to 2.1 eV, and exhibit orbital ordering. Among them, Cr_2_NF_2_ exhibits spin spiral magnetization induced by orbital ordering, where the propagation vector is coupled with the ferroelastic strain of the structure, suggesting the magnetic order can be switched by applying external stress. We also find that the Janus MXene Mo_8_N_4_F_7_O exhibits two distinct ferroelectric phases marked by different orbital orderings, where the energy difference between the two phases can be reduced by applying an external strain or external electric field. Remarkably, the rearrangement of the orbital ordering during transition would also reverse the direction of net magnetism, illustrating the coupling between the strain tensor, electrical polarization, and magnetization. Our results show that orbital ordering can play an important role in coupling the electronic, magnetic, and mechanical properties of 2D nitride MXenes, making nitride MXenes important candidates for potential application in spintronic devices.

## Figures and Tables

**Table 1:** Magnetic moments in sublattice 1 (2) are shown without (with) parentheses. Using a Monte Carlo (MC) simulation, we predicted the magnetic order, Néel temperatures of the AFM lattice structures and transition temperatures of non-collinear magnetic structures. The energy of 2 × 2 supercells with different magnetic configurations (Figure 1a–d) are calculated using DFT and listed with reference to the energy of their respective magnetic ground state.

MXene	Band Gap/eV	Magnetic Moment/µB	Magnetic Order (MC)	Transition Temperature/K	E (AFM1)/meV	E (AFM2)/meV	E (AFM3)/meV	E (AFM4)/meV	E (FM)/meV
V_2_NO_2_	0.78	1.9 (1.2)	AFM3	140	106	131	0	-	44
V_2_NF_2_	0.85	2.7 (2.1)	AFM3	380	87	18	0	814	1304
Cr_2_NF_2_	2.1	3.9 (3.1)	Néel spin spiral	120	394	399	0	340	549
Mo_2_NO_2_	0.95	2.4 (1.9)	AFM3	350	1320	1586	0	1298	2796
Mo_2_NF_2_	1.75	3.9 (3.4)	AFM3	630	1195	1863	0	-	1266
Mn_2_NO_2_	0.20	3.9 (3.4)	Conical spin spiral	110	440	334	336	59	0

“-” indicates DFT calculation failed to converge.

## Data Availability

Data available on request from the authors.
